# Cell-Based Platform for Antigen Testing and Its Application for SARS-CoV-2 Infection

**DOI:** 10.1128/spectrum.00731-22

**Published:** 2022-07-19

**Authors:** Marvin A. Ssemadaali, Sherri Newmyer, Harikrishnan Radhakrishnan, Juan Arredondo, Harold S. Javitz, Satya Dandekar, Parijat Bhatnagar

**Affiliations:** a Biosciences Division, SRI International, Menlo Park, California, USA; b Medical Microbiology and Immunology, University of California Davis, Davis, California, USA; c Education Division, SRI International, Menlo Park, California, USA; University of Prince Edward Island

**Keywords:** cell-based sensors, COVID-19 diagnostics, antigen test, reporter cell, CAR T-cell

## Abstract

We have engineered a cell that can be used for diagnosing active severe acute respiratory syndrome coronavirus 2 (SARS-CoV-2) infections. Isolation of individuals with active infections offers an effective solution for mitigating pandemics. However, the implementation of this practice requires robust infrastructure for rapid and intuitive testing, which is currently missing in our communities. To address this need, we engineered a fast-growing cell line into a cell-based antigen test platform for emerging viruses, i.e., DxCell, that can be rapidly deployed in decentralized health care facilities for continuous testing. The technology was characterized using cells engineered to present spike glycoprotein of SARS-CoV-2 (SARS-CoV-2-Sgp-cells) and Calu-3 host cells infected with competent SARS-CoV-2. Preclinical validation was conducted by directly incubating the DxCell with oropharyngeal swabs from mice infected with SARS-CoV-2. No sample preparation steps are necessary. The DxCell quantitatively detected the SARS-CoV-2-Sgp-cells within 1 h (*P* < 0.02). Reporter signal was proportional to the number of SARS-CoV-2-Sgp-cells, which represents the infection burden. The SARS-CoV-2 DxCell antigen test was benchmarked against quantitative PCR (qPCR) test and accurately differentiated between infected (*n* = 8) and control samples (*n* = 3) (*P* < 0.05). To demonstrate the broad applicability of the platform, we successfully redirected its specificity and tested its sensing function with cells engineered to present antigens from other viruses. In conclusion, we have developed an antigen test platform that capitalizes on the two innate functions of the cell, self-replication and activation-induced cell signaling. These provide the DxCell key advantages over existing technologies, e.g., label-free testing without sample processing, and will facilitate its implementation in decentralized health care facilities.

**IMPORTANCE** Pandemic mitigation requires continuous testing of symptomatic or asymptomatic individuals with rapid turnaround time, and lack of this capability in our community has prolonged pandemic duration leading to obliteration of world economies. The DxCell platform is a cell-based self-replicative antigen test that detects molecular signatures of the target pathogen and can be distributed in small quantities to testing facilities for expansion on site to the desired volume. In this work, we directed this platform to target SARS-CoV-2. Unlike the PCR detection of viral mRNA that requires trained personnel, the DxCell does not require any sample preparation or signal amplification step and introduces an opportunity for a decentralized testing network.

## INTRODUCTION

Unlike the severe acute respiratory syndrome coronavirus 1 (SARS-CoV-1) disease in 2003, in which increased viral transmission (1) was linked to the clinically symptomatic stage, the SARS-CoV-2 infection transmits efficiently from clinically asymptomatic COVID-19 patients to others (2). Asymptomatic viral transmission and pandemic unpreparedness have been responsible for a viral spread that escapes clinical surveillance strategies (3–7), posing a significant obstacle to controlling the outbreak. Aggressive triaging, rapid contact tracing, and testing/retesting of individuals suspected of having the infection are the cornerstones of successful pandemic mitigation. Although difficult to implement on a large scale, monitoring viral transmission through repeated testing followed by immediate quarantine measures helps reduce the reproduction number (R0) to <1 and decrease the spread of COVID-19. The increasing role of younger people as asymptomatic viral carriers suggests the need for more frequent testing at schools and colleges, including a recommendation to test each student regularly (8), with the need for daily testing estimated to require at least 30 million tests per day (9). Therefore, development of rapid, cost-effective, easy-to-use, and more accurate diagnostic technologies for early detection of community infections is critical in controlling the disease spread.

In this study, we report on the development of a cell-based platform technology for antigen testing, DxCell, that uses virus-specific molecular signatures to diagnose individuals with active infections and can be used in both clinically symptomatic and asymptomatic stages. We designed, developed, and validated this platform for the current COVID-19 pandemic. However, the DxCell platform can be rapidly redirected toward any virus. The scientific premise for this technology is based on the T cell’s ability to regulate its activation cascade on interaction with the infected cell and our ability to engineer the T cells into target-specific reporter cells (10, 11). The DxCell uses a fast-growing, immortalized human T-acute lymphoblastic leukemia (T-ALL) cell line (Jurkat cells) as the cellular chassis (doubling time, ~20 h [12]) that is genetically engineered for specificity toward the spike glycoprotein (Sgp) expressed on SARS-CoV-2 virions (i.e., SARS-CoV-2 DxCell). When the SARS-CoV-2 DxCell encounters the Sgp-presenting infected host cell, it activates and synthesizes bioluminescent and fluorescent reporter proteins.

**FIG 1:**
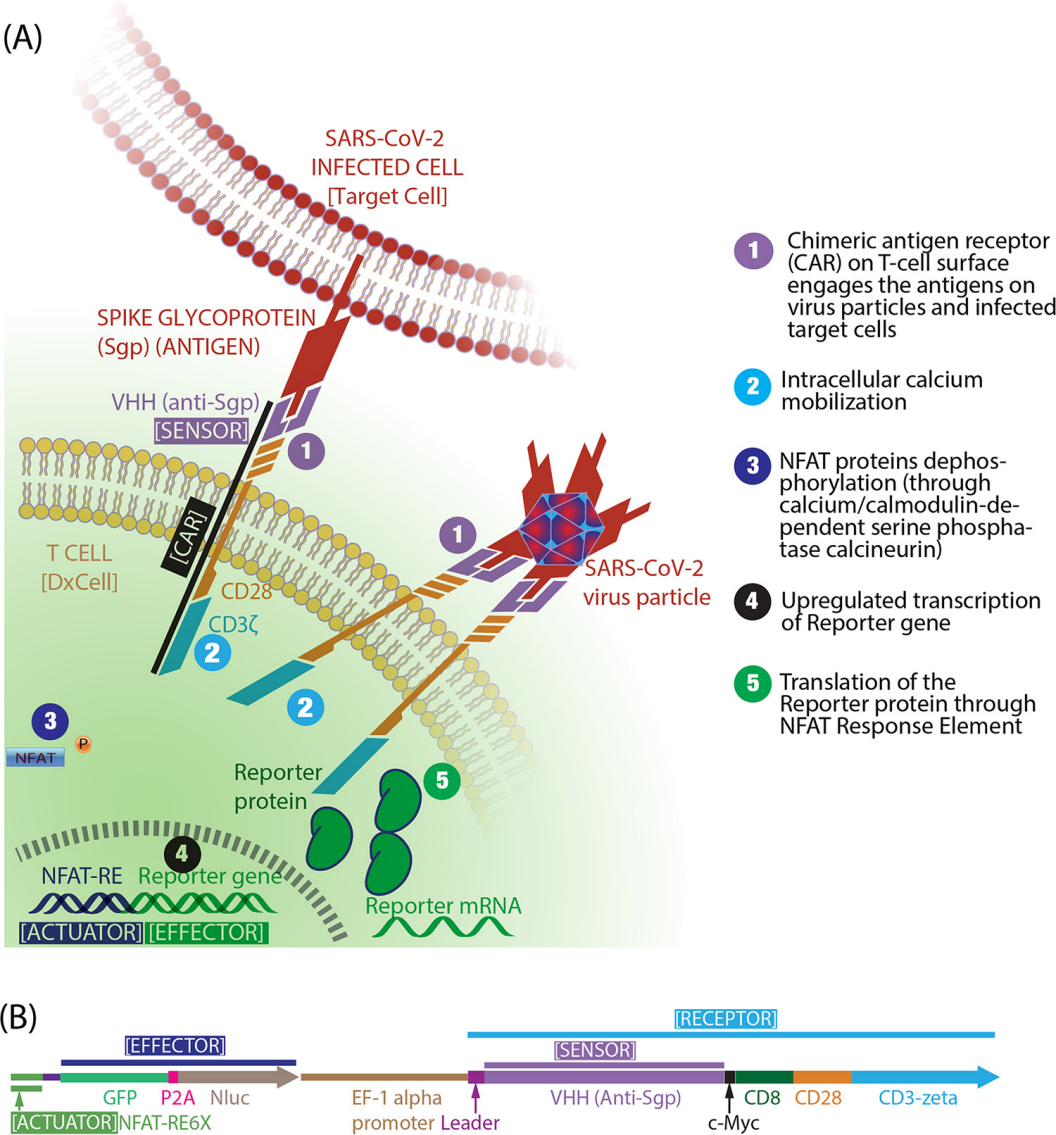
Construct and mechanism of action of the SARS-CoV-2 DxCell. (A) Schematic for DxCell engaging the spike glycoprotein (Sgp) on the host cell infected with SARS-CoV-2 or on the SARS-CoV-2 virus particle. (B) Gene insert for constructing the DxCell.

The schematic in Fig. 1A illustrates the mechanism of the DxCell detecting Sgp antigens on infected cells. The DxCell platform construction uses an artificial cell-signaling pathway composed of two constant and two variable domains in *cis*, the details of which were published earlier (13) and are shown in Fig. 1B. The constant domains provide functionality to the DxCell and include a transmembrane molecule (receptor) that mobilizes the T-cell activation machinery (actuator) to upregulate the desired transgene. The variable domains include a camelid-derived, single-domain heavy chain (VHH) sensor, which is part of the transmembrane receptor that, upon engaging the antigen biomarker, mobilizes the constant domains to synthesize the effector. Both variable domains can be exchanged to impart broad applicability to the DxCell platform. We used the VHH sensor portion to develop a DxCell platform with specificity either for both SARS viruses (SARS-CoV-2 and SARS-CoV-1) or for only SARS-CoV-2 without cross-reactivity to SARS-CoV-1. The effector used in this work is a dual-reporter protein for fluorescence (green fluorescent protein [GFP]) and bioluminescence (NanoLuc [Nluc], Promega, or GFP-2A-Nluc). The third constant domain (secretor), previously reported antigen-specific cell biofactory (13, 14), that assists in the in-situ secretion of the effector is not needed for the DxCell application.

## RESULTS

**FIG 2:**
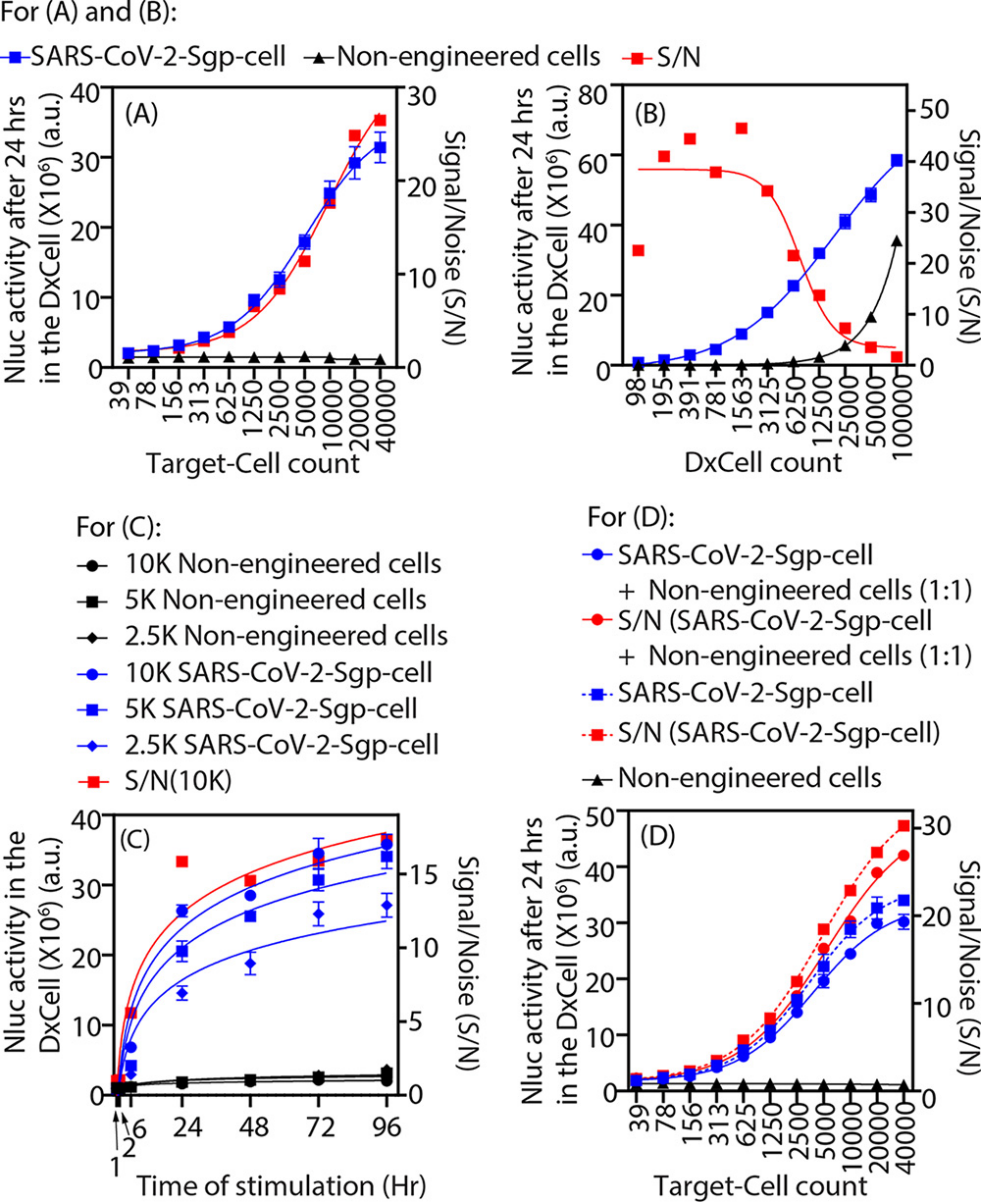
VHH-72 DxCell activation by engineered target SARS-CoV-2-Sgp-cells. The Nluc activity in the VHH-72 DxCell is proportional to the number of target SARS-CoV-2-Sgp-cells (A), proportional to the DxCell count (target cells = 2,500) (B), increased with respect to time and was significantly elevated within 1 h (*P* < 0.02; target cells = 2,500) when stimulated by the target SARS-CoV-2-Sgp-cells versus nontarget cells (C), and not affected in the presence of nontarget cells (D). Nluc activity for all observations was measured using *n* = 4, and error bars indicate ±1 standard deviation (SD). VHH-72 DxCell (12,500 cells) were used for panels A, C, and D.

To demonstrates the functionality of the DxCell platform in a biosafety level 2 (BSL2) laboratory, we used engineered Sgp-expressing cells to simulate target host cells with a SARS-CoV-2 infection. To demonstrate this capability, we engineered the parental HEK293T/17 cell line to stably express the Sgp from SARS-CoV-2 (SARS-CoV-2-Sgp-cells). A non-engineered parental cell line was used as a negative control. The DxCell platform, on the other hand, was engineered to express GFP linked to Nluc through a self-cleavable peptide linker (GFP-2A-Nluc) as target-inducible reporters for quantitative assessment of the infection. The sensor domain of the DxCell platform included the anti-Sgp VHH sequence (VHH-72) (PDB accession code 6WAQ), previously reported to neutralize zoonotic *betacoronavirus* infections (SARS-CoV-2 and SARS-CoV-1) (15). The binding of Sgp on the target via the sensor domain (see schematic in the Fig. 1A) results in the formation of immune synapse. The resulting activation of the DxCell’s transcriptional machinery through intracellular calcium rise (16) quantitatively informs on the infection burden by upregulation of the reporter system (effector). The VHH portion can also be replaced by the variable heavy-light (V_H_-V_L_) portion of the single-chain variable fragment (scFv) of antibodies to provide specificity against any desired antigen. To further demonstrate the ability of the DxCell platform to identify other SARS coronaviruses, we conducted a parallel validation of the VHH-72 DxCell with target SARS-CoV-1-Sgp-cells (expressing the Sgp from SARS-CoV-1). Data in Fig. S1 in the supplemental material show that the SARS-CoV-1 antigen test exhibited a pattern similar to that observed with SARS-CoV-2 in Fig. 2.

The results in Fig. 2A demonstrate that, in addition to the qualitative detection of the infection, the Nluc activity of DxCell provides a quantitative measure that is proportional to the infection burden. The signal-to-noise ratio (S/N), as defined in the figure legend, quantifies the sensitivity of the DxCell. The S/N was ~1.5 at a low infection burden (i.e., ~40 target cells or DxCell to target cell ratio [D:T] = 320:1; *P* < 0.002) and exponentially increased to ~25 at a high infection burden (i.e., at 40,000 target cells [D:T = 1:3.2; *P* < 0.002]). This corroborates computational findings by others (17) regarding the efficacy of cell-based immune targeting at lower D:T ratios.

An important finding in Fig. 2B indicates that a lower DxCell count offers more sensitive detection when stimulated by the same number of targets. This finding is supported by the observation that the S/N is ~1.6 at 100,000 DxCells (D:T = 40:1; *P* < 0.0001) and increases to ~40 at a lower DxCell count (D:T = 1:12.5; *P* < 0.0001). Although this result comes at the expense of longer test durations, the data shows reduced nonspecific interference when lower DxCell counts are used. This is especially important in the context of developing a sensitive diagnostic test where a higher S/N ratio will reduce the possibility of false negatives and false positives.

Figure 2C illustrates the Nluc reporter kinetics of the DxCell when it is stimulated by serially diluted engineered target SARS-CoV-2-Sgp-cells and compared to the control cells. While the Nluc activity was already increased when compared to that of the control sample at 1 h (for 2,500 SARS-CoV-2-Sgp-cells; *P* < 0.02), it continued to increase for at least 96 h. This finding will help in developing an antigen test for mass screening, as the wide readout window will accommodate a large number of patient specimens for assessing the disease penetration in the population. A representative S/N curve is included for 10,000 SARS-CoV-2-Sgp-cells, which increased with the duration of assay (i.e., for D:T of 0.8:1, S/N was ~1 at 1 h [*P* = 0.8] and at ~17 at 96 h [*P* < 0.0001]). While the signal from the DxCell was increased in correlation to a higher infection burden, as represented by the SARS-CoV-2-Sgp-cell count, the kinetics of the test was faster at a lower infection burden. For example, compared to the control cells, the signal was elevated at 1 h when 2,500 SARS-CoV-2-Sgp-cells (*P* < 0.02; S/N, ~1; D:T = 5:1) were used and at 6 h when 5,000 SARS-CoV-2-Sgp-cells (*P* < 0.0001; S/N, ~3.5; D:T = 2.5:1) or 10,000 cells (*P* < 0.0001; S/N, ~5.5; D:T = 1.2:1) were used. This also validates the previous observation that the Nluc activity of the DxCell was proportional to the infection burden (Fig. 2A) and exhibited higher S/N at a limited engagement of the DxCell with the targets (Fig. 2B).

To investigate the precision with which the samples should be prepared and any nonspecific signal from the impurities, we mixed the engineered Sgp-expressing target cells and nonengineered control cells in equal numbers (i.e., 1:1) and serially diluted the cell mixture to stimulate the DxCell. Data in Fig. 2D show that, compared to stimulation by only engineered target cells, the loss in Nluc reporter activity of the DxCell was insignificant (*P* > 0.05 at all D:T). This confirms that there is no contribution of the background noise to the signal generated from the DxCell. As observed earlier (Fig. 2A), the Nluc reporter expression was again proportional to the target cell numbers. These findings demonstrate the sensitivity of the assay with a heightened reporter signal and provide the rationale for applying it to a large number of patient samples with minimal time for sample preparation and purification.

**FIG 3:**
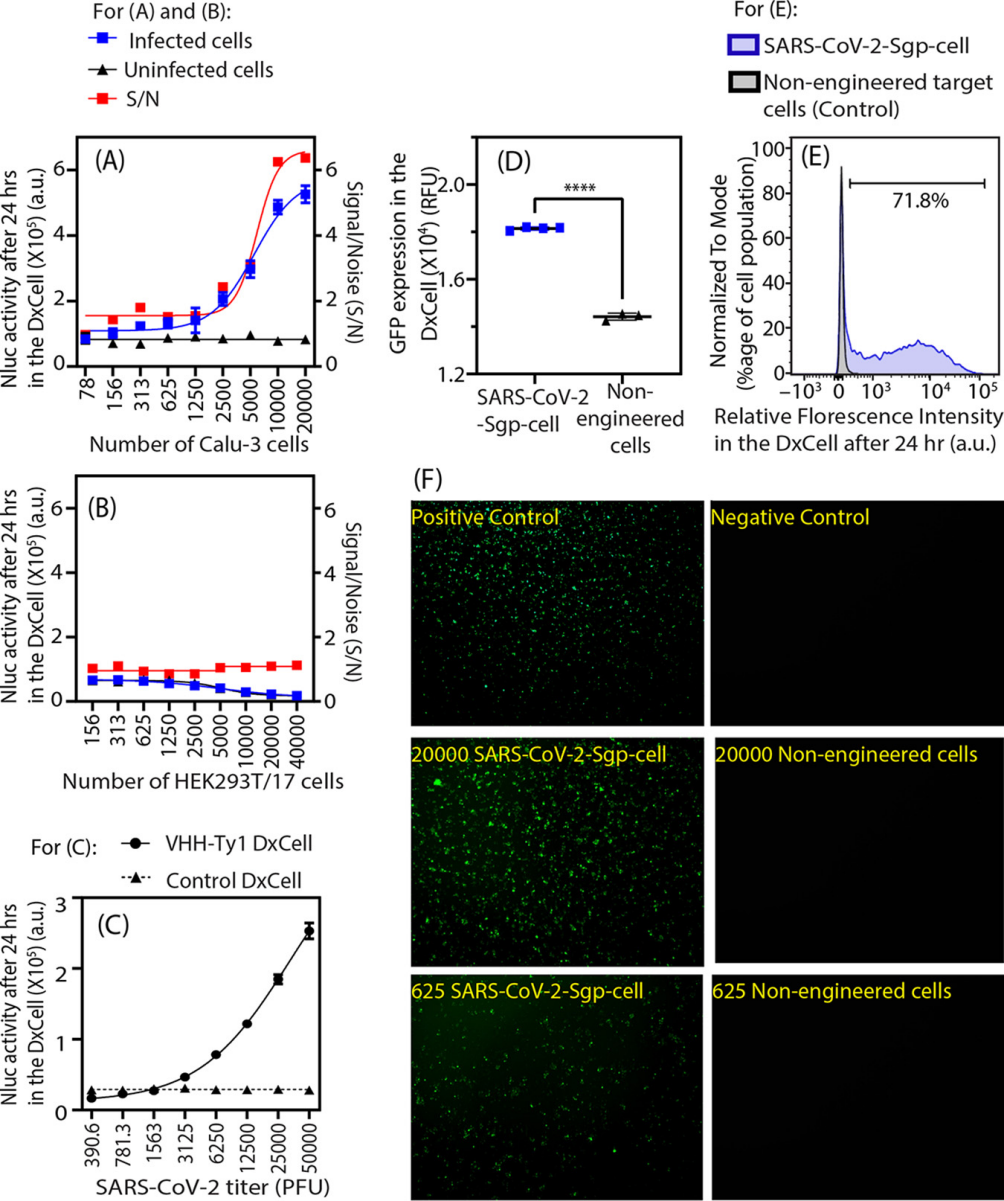
VHH-Ty1 DxCell activation by infected host cells, SARS-CoV-2 virus, or engineered target SARS-CoV-2-Sgp-cells. (A) The Nluc activity in the VHH-Ty1 DxCell is proportional to the number of SARS-CoV-2-infected Calu-3 target cells (*n* = 3). (B) No Nluc activity was observed in the VHH-Ty1 DxCell when SARS-CoV-2-treated HEK293T/17 cells (lacks receptors for SARS-CoV-2) were used as targets. (C) The Nluc activity in the VHH-Ty1 DxCell increased with respect to the number of viral particles present in solution. (D) GFP expression in the VHH-Ty1 DxCell was assessed using plate reader (target cells = 2,500). (E) GFP expression in the VHH-Ty1 DxCell was assessed using flow cytometry. (F) Microscope image panel showing GFP expression in the VHH-Ty1 DxCell at different D:T ratios. All images taken at ×4 magnification. Positive control was prepared by chemically stimulating the DxCell. Unstimulated DxCell was used as negative control. Statistical significance was calculated using the two-tailed Student's *t* test. Nluc activity for all observations was measured using *n* = 4, and error bars indicate ±1 SD. For panels A, B, D, and F, VHH-Ty1 DxCell = 12,500 cells; for panel C, VHH-Ty1 DxCell = 20,000.

We developed and characterized the DxCell technology in a BSL2 containment facility using engineered Sgp-expressing target cells (SARS-CoV-2-Sgp-cells [Fig. 2] and SARS-CoV-1-Sgp-cells [see Fig. S1]). Toward the goal of implementing this technology for detecting SARS-CoV-2, we exchanged the VHH-72 sensor domain of DxCell with the VHH portion of another antibody, VHH-Ty1 (PDB accession code 6ZXN) (18), which is specific to the Sgp of SARS-CoV-2. Figure S2 in the supplemental material describes our efforts to identify the appropriate sensor domain, i.e., VHH-Ty1, among multiple candidates for not cross-reacting with SARS-CoV-1. The rationale for selecting VHH-Ty1 was that we do not expect false positives. Additionally, no cross-reactivity data with other seasonal human coronavirus strains (HKU1, OC43, 229E, and NL63) or emerging variants exist at this time. Results in Fig. 3 show the implementation of this technology with infectious SARS-CoV-2 virus particles (isolate Hong Kong/VM20001061/2020) in a BSL3 containment facility. Initial investigations were conducted by assessing the target-inducible Nluc activity in the VHH-Ty1 DxCell and were later validated by assessing the GFP signal. Negative control was composed of uninfected host cells or a DxCell with abrogated specificity.

Figure 3A demonstrates the quantitative Nluc reporter response of the VHH-Ty1 DxCell. The Nluc activity on engaging Calu-3 host cells infected with competent SARS-CoV-2 virus exhibited a trend similar to that observed with engineered SARS-CoV-2-Sgp-cells in Fig. 2A and was proportional to the infection burden. At a low infection burden, the S/N was ~1 (*P* = 0.83; D:T, ~160:1), which exponentially increased to ~6 at a high infection burden at 20,000 target cells (*P* < 0.0001; D:T = 1:1.6). Figure 3B shows a similar assay conducted by replacing the Calu-3 cells with HEK293 cells. Lack of Nluc expression in the VHH-Ty1 DxCell when stimulated by HEK293T/17 cells treated with infectious SARS-CoV-2 virus correlates with lack of SARS-CoV-2 tropism for targeting HEK293T/17 cells that may not express receptors for virus attachment (19).

To develop an effective antigen test indicative of active infection, we confirmed that the DxCell can detect virus particles. Figure 3C shows the exponential increase of Nluc activity in the DxCell after engaging serially diluted SARS-CoV-2 virion particles ranging from 112,500 PFU to 880 PFU (Nluc activity with respect to the viral titer). This finding is unique, as the lack of costimulatory molecules on virus particles, which are present on the mammalian host cell and are essential for stimulating the T cells (20), challenges the possibility of DxCell successfully detecting target virus particles. The discovery that the DxCell can detect virus particles may be explained by the cross-linking of multiple Sgp-specific VHH (sensor domain) on the DxCell surface assisted by the Sgp of SARS-CoV-2 particles. This initiates the intracellular activation-signaling cascade similar to T cell receptor (TCR)-based activation and has also been reported to activate chimeric antigen receptor (CAR) T cells via multimeric molecules as long as they cross-link the CAR molecules on the T cell surface (21). Findings in Fig. 3C validate that it is, in fact, possible for the virions to directly activate the antigen-specific DxCell.

To further reduce the operational cost of the DxCell platform, we explored the potential of target-inducible GFP expression in the DxCell to assess the status of active infection. The results of experiments performed using the engineered SARS-CoV-2-Sgp-cells (Fig. 3D to F) affirm GFP-based sensing of target cells using the VHH-Ty1 DxCell. This finding reduces the reliance of DxCell technology on a bioluminescent reporter (Nluc), which requires an additional step of substrate addition and contributes toward the cost of the test. To eliminate any chances of infection during the handling of infected specimens, we validated the use of DxCell with samples that underwent heat-inactivation (65°C for 30 min). The Nluc signal from DxCell is significantly elevated (*P* < 0.0001) (see Fig. S3 in the supplemental material) and differentiates between engineered Sgp-expressing cells and nonengineered cells.

**FIG 4:**
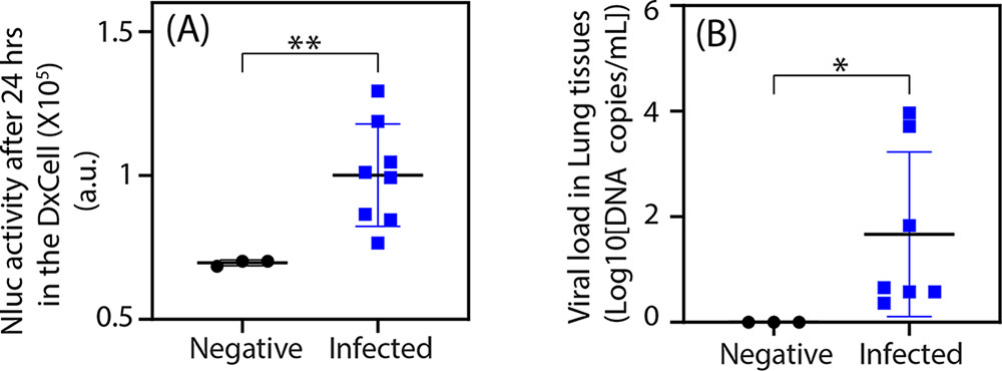
Detection of SARS-CoV-2 infection in mouse oropharyngeal swabs using the VHH-Ty1 DxCell. (A) The scatterplot shows that the VHH-Ty1 DxCell differentiates between infected and negative throat swab samples (*P* < 0.05). (Infected mice, *n* = 8; negative mice, *n* = 3). Each data point represents average Nluc readings from 2 swabs. (B) The scatterplot shows qPCR results for the lung tissues collected from the same mice (lung tissue for one infected mouse was not collected). Statistical significance was calculated using the two-tailed Student's *t* test with Welch’s correction, and error bars indicate ±1 SD.

Finally, we applied the VHH-Ty1 DxCell to detect active infection in specimens obtained from both infected and healthy mice via oropharyngeal swabs. Results validate that the DxCell technology can diagnose active COVID-19 infection via oropharyngeal swabs (Fig. 4A). For the proof-of-principle studies, we assessed the signal from the SARS-CoV-2 DxCell after 24 h. However, our results in Fig. 2C show that the test can be significantly faster with the potential to inform on the active infection within 1 h (for 2,500 SARS-CoV-2-Sgp-cells). Figure 4B shows results obtained by quantitative PCR (qPCR) (as the gold standard) while using mouse lung tissues for comparison with the results from DxCell. These results demonstrate that our diagnostic platform can be rapidly deployed toward emerging viruses and be used with oral specimens without any sample preparation or signal-amplification step.

The impact of this technology goes beyond the current COVID-19 pandemic. To show the expanded applicability of the DxCell platform, we reprogrammed it to detect multiple viruses with the potential to cause future pandemics (22). Figure S4 in the supplemental material shows the feasibility of the DxCell technology against Ebola, Marburg, West Nile virus, Chikungunya, and Nipah using engineered target cells to present virus-specific envelope proteins. A fully matured approach will eliminate the long development periods needed to apply a separate, specific approach for each virus or its variants, which is currently the case for SARS-CoV-2, and rapidly deliver a diagnostic test for any pathogen in less than a month.

Our experiments also confirmed the rapid scalability of the DxCell platform. The doubling time of the VHH-Ty1 DxCell was experimentally determined to be 24.3 (±6.8) h. Our calculations (see Appendix SA and supporting information in the supplemental material) show that if we start with 10 million DxCells, which we have in our current stock (at 10,000 cells/test; including a negative control with an additional 10,000 cells/test), we can scale up to meet the target for 30 million tests/day (9) in less than 20 days.

## DISCUSSION

Between 1940 and 2004, over 335 emerging and reemerging infections were reported in humans (23, 24), which have been responsible for the some of the deadliest pandemics in human history (25). The COVID-19 recession has been the worst global economic catastrophe since the Great Depression of the 1930s. Social distancing has decimated the global economy and has disproportionately affected households at low socioeconomic levels. At its peak, the pandemic forced 4 billion people, roughly half the world’s population, to stay at home. Proactive triaging, rapid contact tracing, and testing/retesting of individuals suspected of having the infection are the cornerstones of effective pandemic mitigation. The daily need for tests has been estimated to be at 30 million/day (9), and in certain subpopulations, testing is recommended every 2 days (8). A technology to triage and identify asymptomatic infected individuals for quarantine will assist in suppressing the disease spread.

In this study, we used the cell-engineering approach to develop a proof-of-concept antigen test for detecting active infection. The use of this antigen test could eliminate shortcomings of detecting genetic materials, which may not truly reflect the status of a patient (26). Other test technologies for SARS-CoV-2 detection have emerged (27–32) that, similar to the DxCell technology, could support decentralized mass screenings of human subjects and identify asymptomatic contagious carriers of SARS-CoV-2. PCR detection of viral mRNA and surface-immobilized antigen testing is being used to identify infections. While these assays have important uses, they have shortcomings (33). For example, PCR tests are highly accurate but require expensive equipment and trained personnel and results take up to 6 h to deliver. The sensitivity of PCR also renders it able to detect residual viral RNA long after the infection has passed, and the patient is no longer contagious (26, 34). The CRISPR-based technologies have specificity and sensitivity for point-of-care diagnostics of viral RNA, but sample preparation is demanding and time-consuming (27, 35).

Cell-based tests for SARS-CoV-2 have been previously reported and commercially available, including a B-cell-based diagnostic technology using the CANARY detection system (36, 37) and bioelectric recognition assay-based biosensor (38). Unlike these cell-based technologies that produce a reporter signal within seconds, the DxCell is a T-cell-based technology that has a slower response time of ~1 to 2 h, but the signal lasts for at least up to 5 days. This makes the DxCell a strong candidate for use in laboratory-based diagnostic tests for mass screening of clinical specimens with low operational burden. The unique advantages of the DxCell over these approaches include the following: (i) a quantitative, label-free, self-replicating, small-footprint diagnostic component that could be readily transported across the globe and expanded on site; (ii) testing/retesting capabilities in decentralized health care facilities for mass screening that does not require complex sample preparation; and (iii) a scalable, practical approach for manufacturability.

We can expect that the DxCell platform will help alleviate current public health and economic crises by implementing a simple, one-step test to measure the viral load, even during subclinical stages and in asymptomatic individuals. Because the test does not involve complex RNA extraction and signal-amplification steps, it can be conducted at local health care facilities. The technology introduces a screening platform that can be redirected toward any emerging virus by exchanging the sensor domain of the DxCell for a sensor specifically aimed at the target antigen. We have also redirected the platform for detecting antibodies and have validated it as a serology test with SARS-CoV-2 sero-positive individuals (manuscript in review). Nevertheless, challenges exist due to lack of infrastructure for cell culture and cryostorage. As with any technology, these will need to be resolved before its widespread use in the clinic. The modularity of the platform also offers the potential to redirect the platform into a therapeutic cell-based platform by exchanging the reporter with a therapeutic protein (13, 14, 39) and forms the foundation of our next steps.

## MATERIALS AND METHODS

### Materials and reagents.

Engineered Jurkat E6-1 (ATCC; catalog number TIB-152) cell lines were maintained in complete RPMI medium (RPMI 1640 [Corning; catalog number 10-040-CV]); 10% were heat-inactivated fetal bovine serum [FBS] [Sigma-Aldrich; catalog number F2442-500ML] and 1× [penicillin-streptomycin solution (Corning; catalog number 30-002-Cl)]. Parental and engineered HEK293T/17 cells (ATCC; catalog number CRL-11268) were cultured in complete Dulbecco modified Eagle medium (DMEM) (DMEM growth medium [Corning; catalog number 10-013-CV] supplemented with 10% FBS [Sigma-Aldrich; catalog number F2442-500ML] and 1× penicillin-streptomycin solution [Corning; catalog number 30-002-Cl]). Calu-3 cells (ATCC; catalog number HTB-55) were cultured in complete Eagle's minimum essential medium (EMEM) (EMEM growth medium [Corning; catalog number 10-009-CV] supplemented with 10% heat-inactivated FBS [Sigma-Aldrich; catalog number F2442-500ML] and 1× penicillin-streptomycin solution [Corning; catalog number 30-002-Cl]). All cells were expanded, and liquid nitrogen stocks were maintained using freezing medium (50% FBS, 40% growth medium, and 10% dimethyl sulfoxide [DMSO]). The SARS-CoV-2 virus culture (BEI Resources, NIH; Hong Kong/VM20001061/2020 [catalog number NR-52282]) was provided by Mary P. Lanier (SRI International). Plasmids encoding different genetic payloads (transfer plasmids) were designed in SnapGene software (GSL Biotech LLC) and subcloned into lentivirus vector plasmid (System Biosciences; catalog number CD510B-1) of piggyBac transposon vector plasmid (System Biosciences; catalog number PB510B-1). Plasmids encoding second-generation packaging genes (psPAX2, catalog number 12260; pMD2.G, catalog number 12259) were received from Didier Trono (Ecole Polytechnique Fédérale de Lausanne, Lausanne, Switzerland) through Addgene (40), while pAdvantage was obtained from Promega (catalog number E1711). piggyBac transposase sequence was provided by Nancy Craig (Johns Hopkins University School of Medicine). All plasmid preparation services (chemical synthesis of DNA insert sequences, subcloning into respective vector backbones, and the amplification) were obtained from Epoch Life Science, Inc. (Missouri City, TX). Transporter 5 reagent (Polysciences, Inc.; catalog number 26008-5) was used to transfect parental HEK293T/17 cells during lentivirus production. The collected lentivirus was transduced into Jurkat cells using Polybrene (abm; catalog number G062). TransIT-2020 transfection reagent (Mirus; MIR5400) was used to transfect piggyBac transposon system plasmids into parental HEK293T/17 cells to engineer stable antigen-presenting cells (APCs) or Sgp-expressing target cells. Puromycin dihydrochloride (Thermo Fisher Scientific; catalog number A1113803) was used for selecting stable cells. Nano-Glo assay (Promega; catalog number N1120) was used to assess the NanoLuc (Nluc) expression activity. Sterile minitip polyester swabs (Puritan; catalog number 25-800-1PD) were used for collection of oropharyngeal swab samples from mice. For RNA preparations from lung tissues, Direct-zol RNA miniprep kit (Zymo Research; catalog number 11-331) and reverse transcription by SuperScript III RT (Invitrogen; catalog number 18080044) were used following the manufacturers’ instructions. TaqMan Universal PCR master mix (Applied Biosystems; catalog number 4305719) was used for reverse transcriptase quantitative PCRs (RT-qPCRs).

### Lentivirus production.

Lentivirus particles were prepared by packaging the transfer plasmid using the second-generation lentivirus system as detailed previously (11).

### Engineering of the DxCell.

The Jurkat E6-1 suspension cell line was engineered with lentivirus particles carrying the genetic payload (Fig. 1) with the artificial cell signaling pathway as detailed previously (11). The sensor domain sequences were selected based on their specificities to the envelope proteins of the respective viruses. The cells were treated with lentivirus in the presence of 8 μg/mL Polybrene. After 48 h, the engineered cells were placed in selection using 0.5 μg/mL of puromycin dihydrochloride. The unmodified parental cell line was also placed under selection as a positive control for cell killing by puromycin. Following selection, cells were expanded as required for different assays and frozen using freezing medium. Sequences from the sensor domain were obtained from the following sources: VHH-72 (PDB accession code 6WAQ) (15), VHH-Ty1 (PDB accession code 6ZXN) (18), S309 antibody (PDB accession code 6WPS) (41), S230 antibody (PDB accession code 6NB8) (42, 43), m396 antibody (PDB accession code 2DD8) (43), CR3022 antibody (PDB accession code 6W41) (44), Ebola virus antibody (PDB accession code 6MAM) (45), Marburg virus antibody (PDB accession code 5JRP) (46), Chikungunya virus antibody (PDB accession code 4GQ9) (47), Nipah virus antibody (PDB accession code 6U1T) (48), and West Nile virus antibody (PDB accession code 1ZTX) (49). All gene sequences were codon optimized before expression using lentivirus plasmids.

### Engineering of target cells with surface expression of virus envelope protein.

The parental HEK293T/17 cells were engineered to stably express the virus envelope protein using the piggyBac transposon system as previously described (10). Two plasmids were designed with the piggyBac transposon vector backbone for cell surface expression of the envelope protein from the respective virus. A monolayer of HEK293T/17 cells was transfected with the transposon plasmid (carrying the gene of interest) and transposase plasmid, in a ratio of 2.5:1, respectively, using TransIT-2020 transfection reagent. After 48 h of transfection, the transfected cells were placed under selection using 0.5 μg/mL of puromycin dihydrochloride. The unmodified parental HEK293T/17 cell line was placed under selection as a positive control for cell killing by the antibiotics. The generated stable cell lines were expanded as required for different assays. Sequences from the gene of interest (envelope proteins) were obtained from the following sources: SARS-CoV-2 (GenBank accession number QHD43416.1, position 1 to 1273), SARS-CoV-1 (GenBank accession number AAP13567.1, position 1 to 1255), Ebola virus (GenBank accession number AAB81004.1, position 33 to 676), Marburg virus (GenBank accession number CAA78117.1, position 19 to 681), Chikungunya (E1E2) virus (GenBank accession number AGX45493.1, position 339 to 1247), Nipah virus fusion gp (GenBank accession number Q9IH63.1, position 27 to 546), and West Nile virus (GenBank accession number AAT11537.1, position 1 to 501). All gene sequences were codon optimized before expression in the piggyBac transposon system.

### Method of use for the DxCell with engineered target cells.

The DxCells (D) and target cells (T) were cocultured at different DxCell to target cell (D:T) ratios in 100 μL of complete RPMI medium in a single well of a 96-well plate. Phorbol myristate acetate (30 nM) and ionomycin (1 μM) solution mixed in 0.5% dimethyl sulfoxide (DMSO) was used for unspecific stimulation of the DxCell (positive control). Negative control comprised of stimulation by 0.5% DMSO. After the specified time in coculture, NanoLuc (Nluc) activity in the DxCell was assessed using the Nano-Glo assay following the manufacturer’s instructions. The Nluc enzyme substrate was diluted in the cell lysis buffer provided with Nano-Glo and added to the cocultures in a 96-well plate for assessing enzyme activity. Following a brief incubation period of 3 min, bioluminescence was read on a microplate reader (Perkin Elmer; EnVision multilabel plate reader, model 2104-0010A). Additionally, GFP expression in the DxCell was assessed using the plate reader, flow cytometry (FACS Aria III; BD Biosciences), and fluorescence microscopy (EVOS FL Auto 2; Invitrogen).

### Method of use for the VHH-Ty1 diagnostic cell with SARS-CoV-2-infected Calu-3 or HEK293T/17 epithelial cells as infected target cells.

Human lung epithelial cells, Calu-3 cells (or HEK293T/17 cells), were cultured in a 6-well plate (1 × 10^6^/well) overnight in complete EMEM and infected as previously described (50). At about 70% confluence, the cells were infected with the SARS-CoV-2 virus culture at an multiplicity of infection (MOI) of 0.05 for 24 h. The infected cells were harvested and then used as the antigen-presenting target cells in coculture experiments. The virus-infected target cells were cocultured at different D:T with the VHH-Ty1 DxCell in 100 μL of complete RPMI medium in a single well of a 96-well plate. Noninfected cells were used as the negative controls. After 24 h of coculture, Nluc activity in the VHH-Ty1 DxCell was assessed using the Nano-Glo assay.

### Method of use for the VHH-Ty1 DxCell with SARS-CoV-2 viral particles.

To test if the VHH-Ty1 DxCell could be used to detect the presence of SARS-CoV-2 viral particles in solution, the DxCells were aliquoted in 100 μL per well of complete RPMI medium containing varying concentrations of serially diluted SARS-CoV-2 viral particles in a 96-well plate (20,000/well). Another DxCell with an abrogated sensor specificity was used as a negative control. The plate was incubated at 37°C for 24 h before Nluc activity was assessed using the Nano-Glo assay.

### Method of use for the VHH-Ty1 DxCell to detect SARS-CoV-2 infections in animal samples.

To check if the DxCell technology can detect active infection in animal samples, we used a mouse model. Briefly, eight (8) heterozygous female K18-hACE c57BL/6J mice [strain 2B6.Cg-Tg(K18-ACE2) 2Prlmn/J; The Jackson Laboratory] were infected intranasally using 50 μL of virus culture (5,000 PFU) per animal, following protocols approved by the Institutional Animal Care and Use Committee at SRI International (#20003), while three (3) mice were not infected (negative controls). Eight days postinfection (DPI-8), the mice were euthanized, and two oropharyngeal swab samples were collected from each animal (51). The swabs were immediately placed into collection tubes containing 500 μL of complete RPMI medium and transported to the BSL3 lab for processing. For each collected swab, the collection tube was vortexed for 10 to 15 s to release cells into the media before the swab was removed from the collection tubes. The collection tube was then centrifuged at 300 g for 5 min to collect cell pellets. The cell pellet was then resuspended into 50 μL of complete RPMI medium. In a 96-well plate, each sample (cells in 50 μL) was cocultured with 50 μL of 25,000 VHH-Ty1 DxCell in complete RPMI medium. The plate was incubated at 37°C for 24 h before Nluc activity was assessed using the Nano-Glo assay.

### Viral RNA extractions and RT-qPCR analysis.

Total RNA was extracted from mouse lung tissue homogenates using the Direct-zol RNA miniprep kit, and reverse transcription was performed using SuperScript III RT, following the manufacturer’s instructions. RT-qPCRs were performed using TaqMan Universal PCR Master Mix, in which samples were processed using the following cycling protocol in the ViiA 7 thermocycler (Applied Biosystems): 50°C for 2 min and 95°C for 10 min, followed by 40 cycles at 95°C for 15 s and 60°C for 1 min. The primer sequences used for RT-qPCR targeted the nucleocapsid (NC) gene of SARS-CoV-2 and are as follows: forward, 5′-GTTTGGTGGACCCTCAGATT-3′; reverse, 5′-GGTGAACCAAGACGCAGTAT-3′; and probe, 5′-/56-FAM/TAACCAGAA/ZEN/TGGAGAACGCAGTGGG/3IABkFQ/-3′. Assay validation was performed using SARS-CoV-2 virus genome to create a standard curve, and the detection limit was determined to be from 5 × 10^6^ to 0.5 viral RNA copies/mL. Results were expressed as log_10_(viral RNA copies/mL).

### Statistical analysis.

GraphPad Prism 9.2.0 (GraphPad Software, Inc.) was used to conduct all statistical analyses. The experimental design and logistical models used for each panel in the figures is described further in the supplemental material.

### Data availability.

All data used to draw conclusions of our work are present in this manuscript and in the supplemental material.
