# Supplementary material for: Cell-Based Platform for Antigen Testing and Its Application for SARS-CoV-2 Infection
Source: Microbiol Spectr. 2022 Jul 19;10(4):e00731-22. doi: 10.1128/spectrum.00731-22 (PMC9430147; doi:10.1128/spectrum.00731-22)
Supplement: Supplemental file 1 — Supplemental material. Download spectrum.00731-22-s0001.pdf, PDF file, 0.3 MB [file spectrum.00731-22-s0001.pdf]

SUPPORTING INFORMATION

Cell-based platform for antigen testing and its application for SARS-CoV-2 infection

*Marvin A. Ssemadaali[1], Sherri Newmyer[1], Harikrishnan Radhakrishnan[1], Juan Arredondo[2], Harold S. Javitz[3], Satya Dandekar[2], Parijat Bhatnagar[1]**

[1]Biosciences Division, SRI International, Menlo Park, CA 94025

[2]Medical Microbiology and Immunology, University of California Davis, Davis, CA 95616

[3]Education Division, SRI International, Menlo Park, CA 94025

**Parijat.Bhatnagar@sri.com*

Running title: Cell-based COVID-19 antigen test

Supplemental information for Statistical analysis. The experimental design and logistical models used for each panel in the figures is described below.

i) *Figure 2 (VHH-72 DxCell activation by engineered target SARS-CoV-2-Sgp-cells).* Statistical analysis for all panels (A–D) was based on an unpaired two-tailed student's t-test with common variance, and the p-value of <0.05 was considered statistically significant. Comparison of data points was done by the false discovery rate (FDR) multiple comparison approach, using the two-stage step-up procedure of Benjamini, Krieger, and Yekutieli with FDR= 1%, and only p-values less than an FDR of 1% were reported. The S/N is calculated as the ratio of the mean Nluc activity in the VHH-72 DxCell when stimulated by the Target Cells (SARS-CoV-2-Sgp-cells) divided by the mean Nluc activity when stimulated by the negative controls (non-engineered parental HEK293T/17 cells). The error bars extend 1 SD above and below the mean and can also be considered as one half-width of an 68% confidence interval for that mean.

ii) *Figure 2A (Nluc activity in DxCell is proportional to the number of Target Cells).* The Nluc activity in the DxCell stimulated by the target (SARS-CoV-2-Sgp-cells) or non-target cells was fitted using a four-parameter logistic model for Nluc given by Nluc(X) = $Nluc_{min}$ + {$Nluc_{max}$ – $Nluc_{min}$}/{1 + 10^[b* (log_{10}[X*] – log_{10}[X])]}; where Nluc(X) is the value of Nluc at X, X is the Target-Cell count, $Nluc_{max}$ is an estimated parameter defining a upper asymptote for Nluc activity, $Nluc_{min}$ is an estimated parameter defining a lower asymptote for Nluc activity, b is a "Hill" parameter defining the slope at the inflection point of the fitted curve, and X_{50} is an estimated parameter representing the X value corresponding to ($Nluc_{max}$ - $Nluc_{min}$)/2.

iii) *Figure 2B (Nluc activity increases with respect to amount of VHH-72 DxCell).* The Nluc activity in the DxCell stimulated by the target (SARS-CoV-2-Sgp-cells) or non-target cells was fitted using a four-parameter logistic model Nluc(X) where X is the DxCell count.

iv) *Figure 2C (Nluc activity in DxCell is a function of duration of stimulation).* The Nluc activity in the DxCell stimulated by the target (SARS-CoV-2-Sgp-cells) or non-engineered cells was fitted using the equation Y = a + b * log_{10}(X), where X is the stimulation time in hours.

v) *Figure 2D (Sensitivity of DxCell is unaffected by the presence of non-target cells).* The Nluc activity in the DxCell stimulated by the target (SARS-CoV-2-Sgp-cells) or non-target cells was fitted using a four-parameter logistic model Nluc(X) where X is the Target-Cell count.

vi) *Figure 3 (VHH-Ty1 DxCell activation by infected host cells, SARS-CoV-2 virus or engineered target SARS-CoV-2-Sgp-cells).* Statistical analysis for all panels as based on an unpaired two-tailed student's t-test with common variance and the p-value of <0.05 was considered statistically significant. Comparison of data points

was done by the false discovery rate (FDR) multiple-comparison approach, using the two-stage step-up procedure of Benjamini, Krieger, and Yekutieli with FDR = 1%, and only p-values less than an FDR of 1% were reported. The S/N is calculated as the ratio of the mean Nluc activity in the DxCell when stimulated by the targets (infected Calu-3 or infected HEK293T/17 cells) divided by the mean Nluc activity when stimulated by the non-infected parental Calu-3 or HEK293T/17 cells. The error bars extend 1 SD above and below the mean and can also be considered as one half-width of an 68% confidence interval for that mean.

vii) *Figure 3A and Figure 3B (Nluc activity in DxCell is proportional to the number of SARS-CoV-2-infected epithelial cells).* The Nluc activity in the DxCell stimulated by the targets (infected Calu-3 or infected HEK293T/17 cells) or non-targets (non-infected Calu-3 or non-infected HEK293T/17 cells) was fitted using a four-parameter logistic model Nluc(X) where X is the infected Target-Cell count.

viii) *Figure 3C (Nluc activity in DxCell is proportional to the number of SARS-CoV-2 viral particles in solution).* The Nluc activity in the VHH-Ty1 DxCell (or DxCell with an abrogated Sensor specificity used as a negative control) stimulated by the SARS-CoV-2 viral particles was fitted using a four-parameter logistic model Nluc(X) where X is the viral particle count (PFU).

ix) *Figure 3D (Detection of GFP expression in the VHH-Ty1 DxCell using a plate-reader).* The scatter plot shows the Nluc activity when the DxCell was stimulated by SARS-CoV-2-Sgp-cells or parental HEK293T/17 cells. An unpaired student's two-tailed t-test was used to determine the statistical difference between the two samples, assuming a common variance and a *p*-value of 0.05.

x) *Figure 3E (Detection of GFP expression in the VHH-Ty1 Diagnostic Cell using Flow-cytometry).* Expression of GFP (Relative Fluorescence Intensity) in the DxCell is

analyzed using Flow-Jo software 10.8.0 (BD Biosciences) and the cell counts are normalized to the respective cell type.

xi) *Figure 4A (Detection of SARS-CoV-2 infection in mouse oropharyngeal swabs using the VHH-Ty1 DxCell).* A scatter plot (confidence interval of 95%) shows the difference between throat swabs collected from SAR-CoV-2-infected mice and non-infected mice, using an unpaired two-tailed student's t-test with Welch's correction and assuming Gaussian distribution.

xii) *Figure 4B (Diagnosis of SARS-CoV-2 infection in mouse lung tissues by qPCR).* A scatter plot (confidence interval of 95%) shows the difference between viral loads of mouse lungs tissues collected from SAR-CoV-2-infected mice and non-infected mice, using an unpaired two-tailed students' t-test with Welch's correction and assuming Gaussian distribution.

xiii) *Figure S1 (VHH-72 DxCell activation by engineered target SARS-CoV-1-Sgp-cells).* Statistical analyses for SARS-CoV-1 related investigations reported in all panels (A–D) were similar to that employed for SARS-CoV-2 in the respective panels of Figure 2.

xiv) *Figure S2 (Screening of different DxCells with specificity towards SARS-CoV-2 and SARS-CoV-1).* The Nluc activity in the DxCell stimulated by the target (SARS-CoV-2-Sgp-cells or SARS-CoV-1-Sgp-cells) or non-target cells was fitted using a four-parameter logistic model Nluc(X) where X is the Target-Cell count.

xv) *Figure S3 (Detection of heat-inactivated Target Cells by the VHH-Ty1 DxCell).* A scatter plot (confidence interval of 95%) shows the difference in Nluc expression signal in the DxCell by heat-inactivated engineered Sgp-expressing Target Cells (by air or beads at 65°C) versus non-engineered cells, using an unpaired two-tailed

students' t-test, assuming Gaussian distribution and that both sample means have the same standard deviations.

xvi) *Figure S4 (Development of different DxCells with specificity to other emerging viruses).* The Nluc activity in the DxCell stimulated by different Target Cell types (engineered Target Cells displaying the glycoproteins from (A) Ebola virus; (B) Marburg virus; (C) Chikungunya virus; (D) Nipah virus; and (E) West-Nile virus) or non-target (non-engineered parental HEK293T/17) control cells was fitted using a four-parameter logistic model Nluc(X), where X is the Target-Cell count.

SUPPLEMENTAL FIGURES

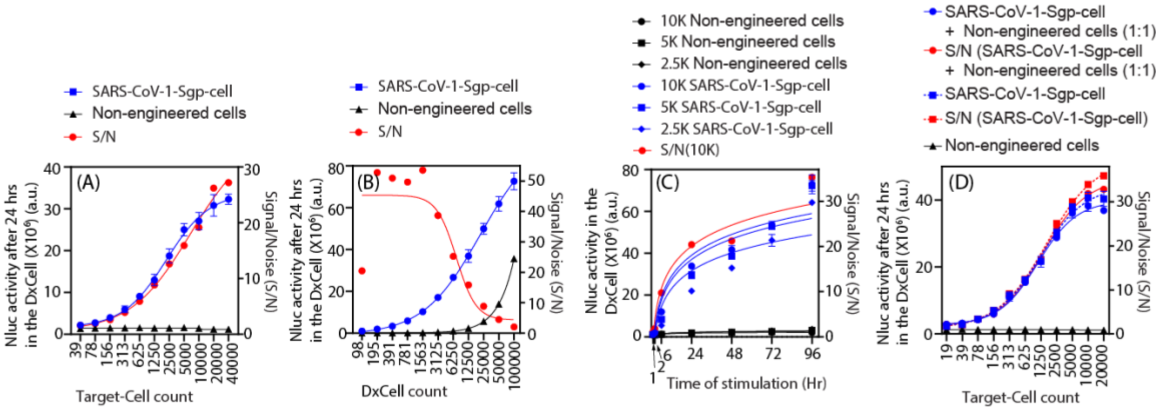

Figure S1. VHH-72 DxCell activation by engineered target SARS-CoV-1-Sgp-cells. The Nluc activity in the VHH-72 DxCell is **(A)** proportional to the number of target SARS-CoV-2-Sgp-cells. **(B)** proportional to the DxCell count (Target Cells = 2,500). **(C)** increased with respect to time when stimulated by the target SARS-CoV-1-Sgp-cells versus non-target cells (Target Cells = 2,500). **(D)** not affected in presence of non-target cells. Nluc activity for all observations was measured using n = 4, error bars indicate ±1 SD and can also be considered as one half the width of an 68% confidence interval for that mean. VHH-72 DxCell (12,500 cells) was used for (A), (C), and (D).

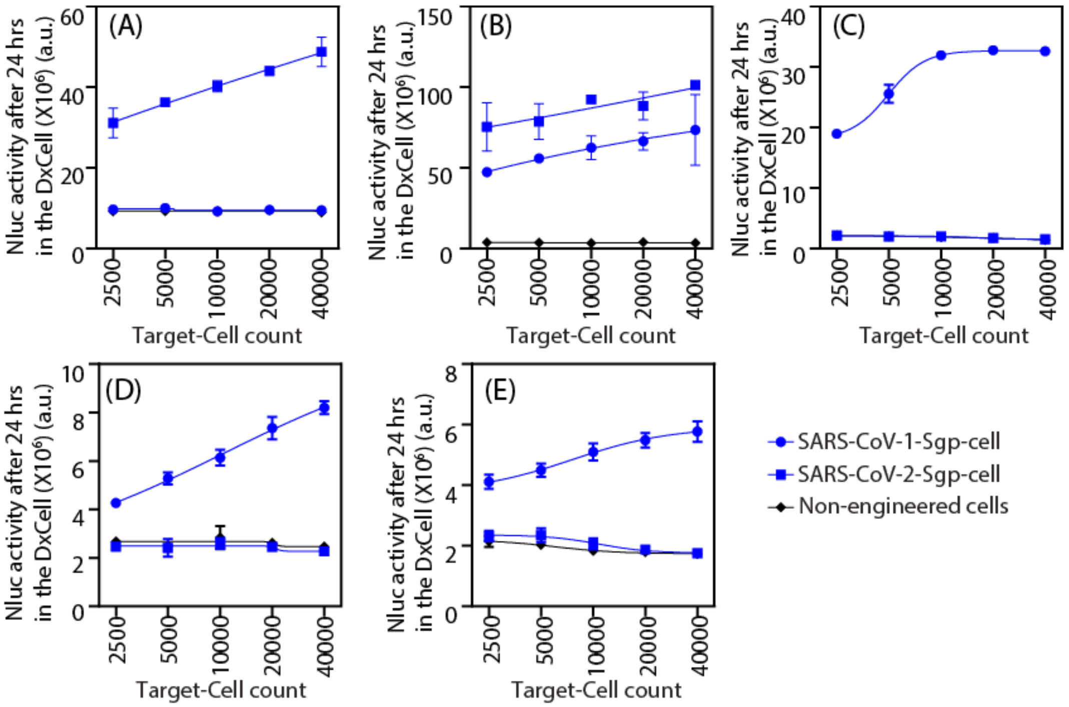

Figure S2. Screening of different DxCells with specificity towards SARS-CoV-2 and SARS-CoV-1. The Nluc activity in different DxCell types is proportional to the number of Target Cells (SARS-CoV-2-Sgp-cells or SARS-CoV-1-Sgp-cells). The Sensor sequences were obtained from **(A)** VHH-Ty1, **(B)** S309 antibody, **(C)** S230 antibody, **(D)** m396 antibody, and **(E)** CR3022 antibody. In all experiments, DxCell = 12,500 and Nluc activity for all observations was measured using n = 4; error bars indicate ±1 SD and can also be considered as one half the width of an 68% confidence interval for that mean.

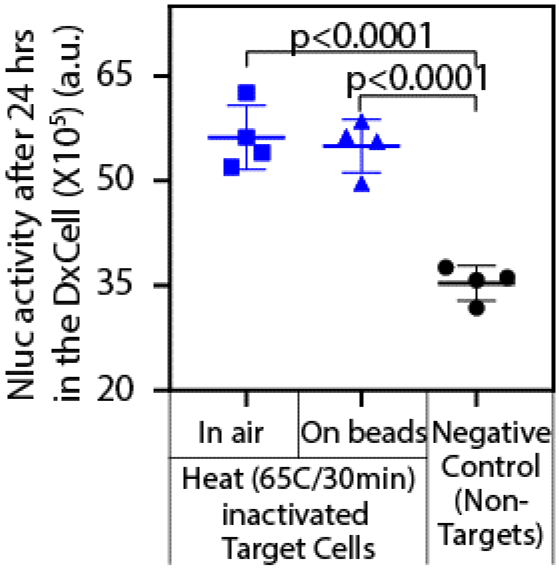

Figure S3. Detection of heat-inactivated Target Cells by the VHH-Ty1 DxCell. A scatter plot shows the Nluc activity in the VHH-Ty1 DxCell (12,500 cells) upon interacting with engineered Sgp-expressing Target Cells (SARS-CoV-2-Sgp-cells = 5,000 cells) heat-inactivated in air or on beads at 65°C vs. non-engineered cells. Nluc activity for all observations was measured using n = 4; error bars indicate ±1 SD.

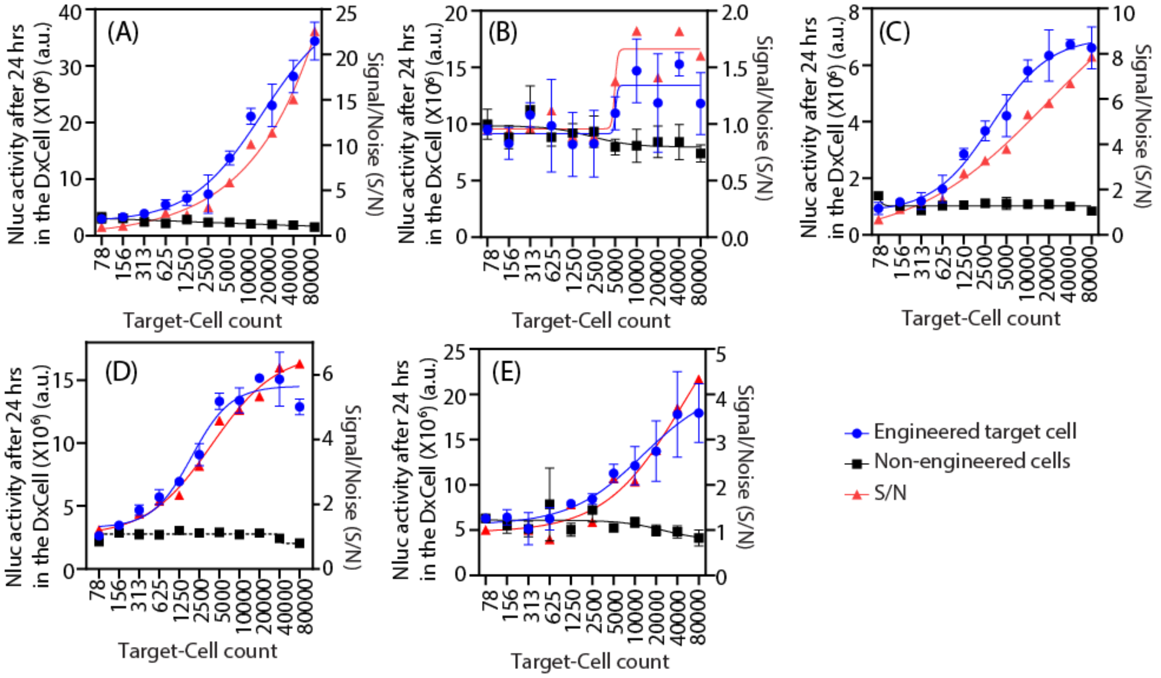

Figure S4. Development of different DxCells with specificity to other emerging viruses. The Nluc activity in the different types of DxCells is specific to their targets and is proportional to the number of Target Cells engineered for surface expression with envelop proteins. The sequences for the envelop proteins were obtained from **(A)** Ebola virus; **(B)** Marburg virus; **(C)** Chikungunya virus; **(D)** Nipah virus; and **(E)** West-Nile virus. In all experiments, DxCell = 12,500 and Nluc activity for all observations was measured using n = 4; error bars indicate ±1 SD and can also be considered as one half the width of an 68% confidence interval for that mean. PDB numbers are provided for the Sensor domain of the respective DxCell.

APPENDIX A: Calculation showing how the DxCell expands to meet 30 million tests/day

No. of DxCells needed for testing the specimen = 10,000

No. of DxCells for the testing the control = 10,000

Therefore, no. of DxCells needed for each test panel = 10,000 + 10,000 = 20,000

No. of DxCells needed to produce 30 million tests/day = 30 million X 20,000 = $6 X 10^{11}$

No. of DxCells (stock) needed to maintain continuous supply of $6 X 10^{11}$ DxCells per day $= (6 X 10^{11}) X 2 = 12 X 10^{11}$

No. of DxCells currently in possession = 10^{7}

Calculation for the no. of DxCells doublings (N) needed

$10^{7} X 2^{N} = 12 X 10^{11}$

N = 16.87

Time for each doubling period of the DxCell = 24.3 ± 6.8 hours

Time for DxCell stock scale up = (24.3 ± 6.8) hours X 16.87 = 409.94 ± 114.7 hours = **17 ± 4.7 days**
